# 608 Geomapping and Spatial Analysis to Examine Socioeconomics, the Built Environment, and House Fires

**DOI:** 10.1093/jbcr/iraf019.237

**Published:** 2025-04-01

**Authors:** Lucy Wibbenmeyer, Alba Paulsen, Siri Pothula, Colette Galet, gilsu Pae

**Affiliations:** University of Iowa; University of Iowa; University of Illinois at Chicago; University of Iowa; University of Iowa

## Abstract

**Introduction:**

House fires result in devastating injuries that are costly to individuals and their communities. Low socioeconomic communities experience a disproportionate share of house fires. We noticed an increasing trend in admissions related to house fires at our burn center. Applying Geographic information system (GIS) and spatial analysis, we examined the relationship between residential fires and socioeconomic variables included in well-established vulnerability indices, the area deprivation index (ADI) and social vulnerability index (SVI).

**Methods:**

This was a retrospective cohort study. Addresses of house fires occurring in our state and resulting in admissions to our burn center were obtained from our burn registry. Socioeconomic and built environment datasets were collected from the 2020 Census Bureau data and transformed into quartiles. The residential addresses of fires were geocoded and spatially merged at the census tract level with the collected Census data. Spatial binary logistic regression models were used to estimate the association between social vulnerability factors and multiple serious house fires. Distance to the burn center was included as a covariate.

**Results:**

The ADI was more closely associated with residential fires than the SVI. Separate logistic regression models for each predictor suggest that most social vulnerability factors included in ADI and SVI were associated with multiple residential fires: family poverty, unemployment, low education, household income, single parent ratio, house values, Black resident ratio, Hispanic resident ratio, monthly gross rent, no vehicle ratio, home ownership, and overcrowded homes. A spatial multivariable logistic regression model showed that the odds of experiencing multiple serious residential fires were higher in the 4th quartiles of the ADI (higher deprivation; OR = 5.9), the Black resident ratio (OR = 4.9), and the mobile home ratio (OR 2.5) compared to the 1st quartiles (p < 0.05).

**Conclusions:**

Serious house fires are associated with increasing social disparities. Our results suggest that identifying census tracts with higher ADI, Black resident ratios, and mobile home ratios can improve focused multi-stakeholder community interventions in our state. Further studies are warranted to build a more precise model for house fire prevention.

**Applicability of Research to Practice:**

GIS and spatial analysis can be used to explore the relationship between socioeconomic and physical environment variables and serious housefires. This knowledge could be used to inform a community intervention to decrease these deadly and expensive fires.

**Funding for the Study:**

Study was partially funded by Injury Prevention Center

